# An Artificial Intelligence Exercise Coaching Mobile App: Development and Randomized Controlled Trial to Verify Its Effectiveness in Posture Correction

**DOI:** 10.2196/37604

**Published:** 2023-09-12

**Authors:** Han Joo Chae, Ji-Been Kim, Gwanmo Park, David Michael O'Sullivan, Jinwook Seo, Jung-Jun Park

**Affiliations:** 1 Department of Computer Science and Engineering Seoul National University Seoul Republic of Korea; 2 Division of Sports Science Pusan National University Busan Republic of Korea

**Keywords:** home workout, mobile assistant, deep-learning, posture correction, physical activity, exercise, social distance, COVID-19, mobile device, workout

## Abstract

**Background:**

Insufficient physical activity due to social distancing and suppressed outdoor activities increases vulnerability to diseases like cardiovascular diseases, sarcopenia, and severe COVID-19. While bodyweight exercises, such as squats, effectively boost physical activity, incorrect postures risk abnormal muscle activation joint strain, leading to ineffective sessions or even injuries. Avoiding incorrect postures is challenging for novices without expert guidance. Existing solutions for remote coaching and computer-assisted posture correction often prove costly or inefficient.

**Objective:**

This study aimed to use deep neural networks to develop a personal workout assistant that offers feedback on squat postures using only mobile devices—smartphones and tablets. Deep learning mimicked experts’ visual assessments of proper exercise postures. The effectiveness of the mobile app was evaluated by comparing it with exercise videos, a popular at-home workout choice.

**Methods:**

Twenty participants were recruited without squat exercise experience and divided into an experimental group (EXP) with 10 individuals aged 21.90 (SD 2.18) years and a mean BMI of 20.75 (SD 2.11) and a control group (CTL) with 10 individuals aged 22.60 (SD 1.95) years and a mean BMI of 18.72 (SD 1.23) using randomized controlled trials. A data set with over 20,000 squat videos annotated by experts was created and a deep learning model was trained using pose estimation and video classification to analyze the workout postures. Subsequently, a mobile workout assistant app, Home Alone Exercise, was developed, and a 2-week interventional study, in which the EXP used the app while the CTL only followed workout videos, showed how the app helps people improve squat exercise.

**Results:**

The EXP significantly improved their squat postures evaluated by the app after 2 weeks (Pre: 0.20 vs Mid: 4.20 vs Post: 8.00, *P*=.001), whereas the CTL (without the app) showed no significant change in squat posture (Pre: 0.70 vs Mid: 1.30 vs Post: 3.80, *P*=.13). Significant differences were observed in the left (Pre: 75.06 vs Mid: 76.24 vs Post: 63.13, *P*=.02) and right (Pre: 71.99 vs Mid: 76.68 vs Post: 62.82, *P*=.03) knee joint angles in the EXP before and after exercise, with no significant effect found for the CTL in the left (Pre: 73.27 vs Mid: 74.05 vs Post: 70.70, *P*=.68) and right (Pre: 70.82 vs Mid: 74.02 vs Post: 70.23, *P*=.61) knee joint angles.

**Conclusions:**

EXP participants trained with the app experienced faster improvement and learned more nuanced details of the squat exercise. The proposed mobile app, offering cost-effective self-discovery feedback, effectively taught users about squat exercises without expensive in-person trainer sessions.

**Trial Registration:**

Clinical Research Information Service KCT0008178 (retrospectively registered); https://cris.nih.go.kr/cris/search/detailSearch.do/24006

## Introduction

A recent study on the relationship between physical activity and the risk of COVID-19 has shown that engaging in the recommended levels of physical activity decreases the likelihood of SARS-CoV-2 infection, severe COVID-19 illness, and COVID-19–related death [1]. However, social distancing and lockdown after the outbreak of COVID-19 have resulted in a vigorous decrease in physical activity [2]. Since such reduction in physical activity could increase risks of not only COVID-19 but also cardiovascular diseases (eg, obesity, hypertension, diabetes, and metabolic syndrome) and even sarcopenia [3-7], physical activity is highly recommended during the confinement through the performance of aerobic, strength, flexibility, and balance exercises [8]. Body weight exercises, such as squats, sit-ups, and push-ups, are some of the best options because they are easy to perform at home without additional equipment and involve various joints and muscle movements [9].

Maintaining proper posture is vital to reap meaningful benefits from workouts. An incorrect posture can cause abnormal muscle activation or apply unwanted pressure on the body’s joints, leading to less effective session results or even injuries [10,11]. Unfortunately, avoiding incorrect postures is not an easy task for nonexperts. It is difficult for people to view their own bodies from an objective perspective, especially while exercising. A widely accepted solution is to have someone else watch and provide feedback. However, nonexperts often do not have proper knowledge of correct postures, and hence, cannot provide helpful feedback. But experts can provide valuable feedback and increase the quality of workout sessions; however, they are in short supply and are often expensive. Moreover, under the current COVID-19 pandemic, in-person meetings and exercise sessions should be avoided.

Remote coaching is one of the most effective solutions for this problem. The recent surge in sales of home-fitness gear [12] supports such a change in exercise trends. Some people purchase equipment and subscribe to web-based services. For example, Nike+ Kinect Training (Nike) [13] is a fitness game for Xbox 360 (Microsoft Corp) that uses Microsoft Kinect, a depth-sensing camera device, and Weelo (Alyce Healthcare) [14] is a web subscription service that analyzes training forms using a laptop. Several studies have been conducted on computer-assisted postural correction. For example, Chen et al [15] built a system that analyzed workout posture using pose estimation; however, they used geometric methods and dynamic time warping using small amounts of data. Han et al [16] proposed using deep neural networks to analyze skeleton data extracted using Microsoft Kinect but did not actually show the implementation of the results. All these systems either require specific equipment or use heuristic methods that are difficult to generalize.

Some of the recent studies used deep learning–based exercise programs. Liao et al [17] developed a system that recognized rehabilitation exercises and Soro et al [18] used the deep learning model to classify several different exercises. However, none of them provided feedback about correct postures. In contrast, this study tried to imitate experts’ visual judgments on correct exercise postures learned from years of experience through deep learning, which is well-known for extracting patterns from image and video data. Furthermore, while various non–face-to-face exercise methods using apps or services [12-16] have surged during the prolonged COVID-19 outbreak, studies on the effects of these methods are insufficient. Quantitative evaluations of posture correction and muscle strength improvements are limited as most studies focus on the qualitative effects of behavioral changes or weight loss [19].

This study aimed to use deep neural networks to design and develop a personal workout assistant capable of providing feedback on squat postures using only mobile devices such as smartphones. In the first part of this study, a squat video data set was created and a deep learning model using a combination of pose estimation and video classification was trained to analyze workout postures. In the second part, a mobile workout assistant app was developed and an interventional study was conducted to show how the app helps people improve squat exercise, in contrast to simply following videos over the internet.

## Methods

### Part 1: Deep Learning Model

#### Data Collection

**Figure 1:**
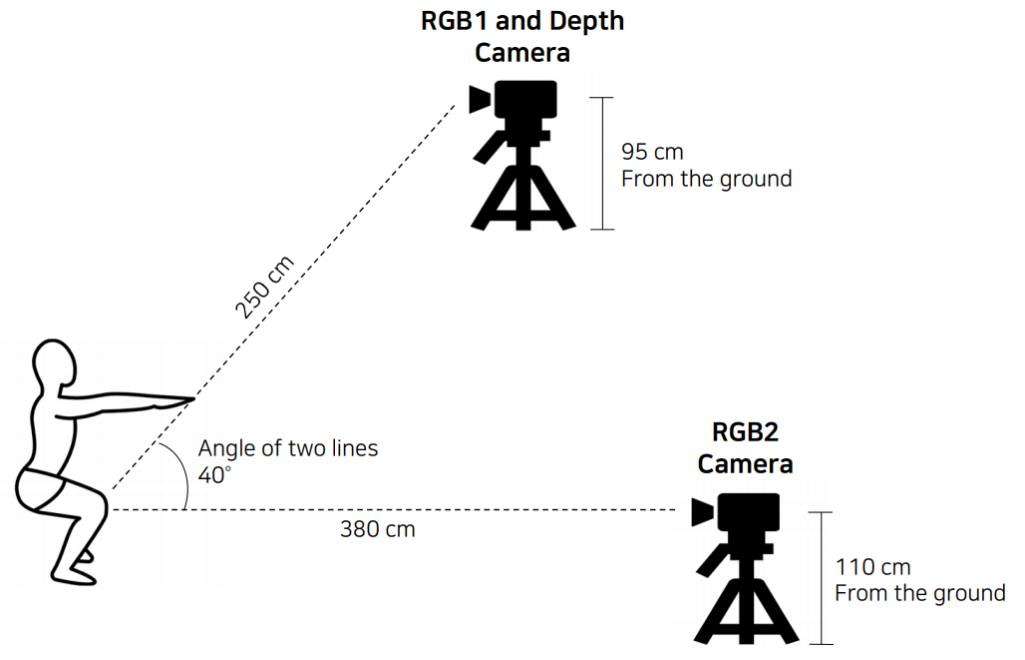
Camera placements during data collection. RGB: red green blue.

In the first part of the study, a squat posture data set was collected from participants to train the neural network model for a mobile app. Chosen participants were adults with no diagnosed musculoskeletal conditions and no pain in the ankles, knees, or lower back for the past 6 months. Those who could not perform the normal range of motion (ROM) for physiological reasons were excluded. A total of 52 participants (36 men and 16 women) were recruited, comprising a mix of 24 novices with little or no experience doing squats and 28 experts with extensive knowledge of correct workout postures. After receiving sufficient explanation about the experiment, participants signed a consent form. Each participant performed less than 100 squats in a single session and less than 200 squats in a single day, with sufficient breaks in between to minimize fatigue and possible injuries. Each participant performed an average of 400 squats, with each squat recorded individually. A Microsoft Kinect One was placed in front of the participant at a distance of 2.5 m and height of 0.95 m to capture the depth and red green blue (RGB) images; a laptop was placed at a distance of 3.8 m and height of 1.1 m to capture RGB images in a diagonal direction using its webcam (Figure 1). Both devices recorded at 30 fps. The EyesWeb program was used to synchronize the recording devices. Three workout experts were present during the recording to determine the correctness of each squat. Any disagreements were resolved by reviewing the video recordings. The problematic body parts were later labeled by experts through video analysis.

#### Data Preparation

Of the collected data, only RGB videos recorded from the front were used to train the deep learning model in this study. This section describes the preprocessing steps performed to transform the videos into trainable data.

**Figure 2:**
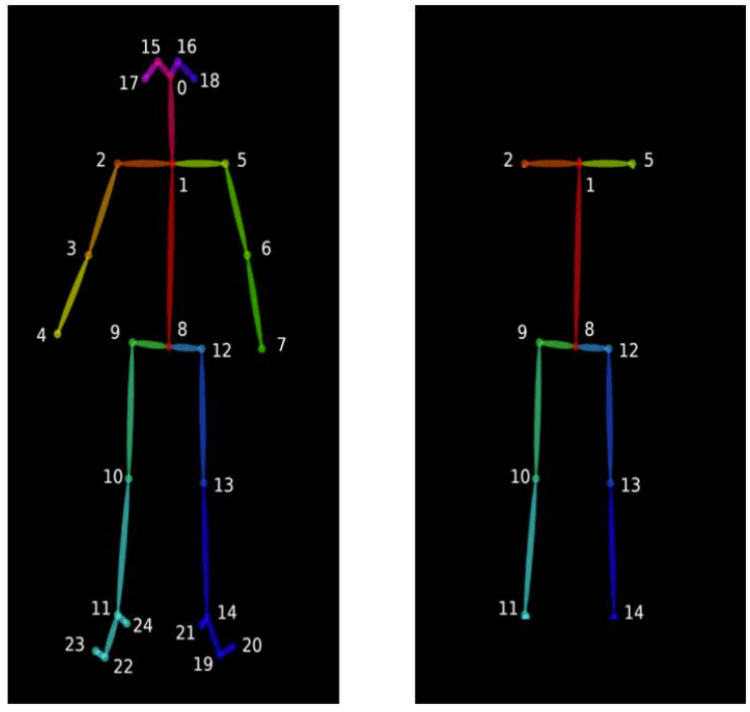
On the left: raw key point output. On the right: key points used for the training.

The videos recorded from the front camera were trimmed to accelerate the pose estimation process. OpenPose [20] was used to extract 2D skeleton key points from the videos, but only a certain portion of the output (see Figure 2) was used as some parts of the body, such as arms, head, and feet, do not have a significant impact on the correctness of the squat posture.

To normalize the key point data, a reference frame was selected from the early portion of the videos to ensure the highest chance that the target was upright and standing straight. Once the reference frame was chosen, a transform matrix that would map the coordinates of the hip joint key point to (0, 0) and the length of the torso to 1 in the reference frame was found and uniformly applied to all frames, ensuring that the coordinates of the key points would fall within a similar bound of values, thus normalizing data points from individuals with different heights. To mitigate the inevitable errors from pose estimation, a Gaussian filter was applied with an SD of 1 for each key point sequence, which smoothed out the movement of each key point, suppressing any unwanted sudden jumps. Finally, the per-frame changes in the normalized key point positions were calculated and used as input to the classification model. For example, if a person was detected in k frames of the video, the size of the input tensor to the model k was –1×20; if the person did not move throughout the video, the resulting input to the classification model was a tensor with all zeros.

The preprocessed key point data were then used as the input for the deep neural network, which classifies them as either correct or incorrect postures.

#### Architecture

**Figure 3:**
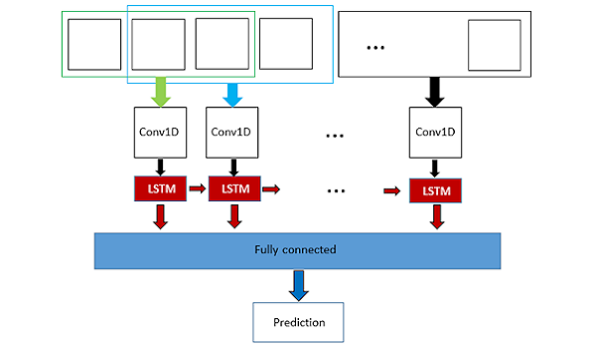
Proposed model architecture. LSTM: long short-term memory; Conv1D: 1D convolution layer.

The task of classifying squat postures can be viewed as a branch of the action recognition task in which videos are classified according to the actions performed by humans. It is better to understand the whole action instead of per-frame information; therefore, the temporal aspect of the data is crucial for the task. The concepts from long-term recurrent convolutional network [21] and convolutional 3D network [22] were combined to design a model with both temporal convolution layers and long short-term memory (LSTM) blocks. The architecture consisted of three 1D convolution layers, 1 bidirectional LSTM layer, and 1 fully connected layer, followed by a softmax layer. The convolution layers had temporal kernel depths of 5, 3, and 3. In other words, in the first convolution layer, 5 consecutive frames were convoluted to create new features; in the second and third convolution layers, 3 consecutive outputs were convoluted together. The number of filters on the convolution layers was 32, 64, and 64. The bidirectional LSTM block had 64 outputs, whereas the fully connected layer had 32 outputs. The model was trained using the Adam optimizer with a learning rate of 0.003 and a dropout rate of 0.3. These parameters were determined using a grid search, which yielded the highest validation accuracy of 0.9866. Figure 3 shows a simplified illustration of the proposed model architecture.

#### Evaluation

The proposed deep learning model was evaluated using the test set described in section 3; the results are reported in Table 1. The model achieved an accuracy of 0.8498, a precision of 0.8713, and a recall of 0.8394. In addition to the model described in Section 4, models without convolution layers and without the LSTM blocks were trained and evaluated. As no suitable baseline performance for squat postures was found in prior work, the results of the Auto-ML Video Intelligence service of Google Cloud [23] were used as the baseline; although not designed for this task, it is still capable of classifying videos. The results are presented in Table 1. The combination of LSTM blocks and temporal convolution layers exhibited the highest performance with a test accuracy of 85%. Convolution-only methods performed better (82% accuracy) than LSTM-only methods (78% accuracy), implying that the benefits of the temporal convolution layer are greater than those of the LSTM blocks. Google AutoML video intelligence service had the lowest accuracy (69%). The test accuracies were significantly lower than the training and validation accuracies for all 3 versions of the model, possibly due to overfitting on all 3 occasions, or it could mean that a large amount of the test set data is quite different from the data in the training set.

**Table 1:** Accuracy of the proposed model.

Model	Training accuracy	Validation accuracy	Test accuracy
LSTM^a^ + Convolution	0.9929	0.9866	0.8498
LSTM only	0.8712	0.8532	0.7613
Convolution only	0.9546	0.9441	0.8203
Google Auto-ML	—^b^	—	0.6880

^a^LSTM: long short-term memory.

^b^Not available.

### Part 2: Mobile Workout Assistant

#### Overview

After testing the proposed model, a mobile app, Home Alone Exercise (HALE), was designed and developed to assist users in learning squat exercises. In addition, an interventional study was conducted to demonstrate the effectiveness of the app by analyzing how the participants improved their exercises over time in terms of performance and joint ROM. This study was retrospectively registered on the Clinical Research Information Service (KCT0008178). The study design was unmodified after it began.

#### App Design

HALE was implemented for mobile devices, comprising 2 components: a server and a mobile app client. The server receives a single squat workout video each time, analyzes the posture through the pipeline, and responds with the classification score (the output of the softmax layer) along with the received video, but with the extracted key points rendered on top. The client allowed users to record their squat posture and send the recording to the server for analysis. The recording starts 3 seconds after the user touches the “start” button to give them time to position themselves correctly. The recording ended automatically after a certain amount of time configured by the user, and the recorded video was sent to the server. Visual and auditory cues are provided at the start and end of the recording session. When the client received a response from the server, the recorded video was shown to the user along with the extracted key points, and posture correctness was checked—such feedback was designed to guide the user to monitor one’s previous squats (eg, balance, joint angles, etc) so that they could learn the correct posture through self-discovery [24]. The user could configure the classification score threshold to adjust the difficulty of the workout session. The app’s best results were obtained when the device was placed at a height of approximately 130 cm (or just below the chest height) and far enough from the user’s workout spot to capture the user’s entire body. The user must face the camera, just like in the videos used in training. The server was implemented using Python and TensorFlow (Google Brain), and the client was implemented for Android devices using Android Studio (Google and JetBrains).

#### Recruitment

The volunteers for the test were recruited through a web-based forum on the Pusan National University web page. Preliminary screening was conducted over the phone using the Physical Activity Readiness Questionnaire for Everyone [25] to select individuals without any health issues. They were then asked whether they have learned squat exercise before and selected ones without any squat exercise experience. Volunteers who passed this preliminary selection underwent secondary in-person screening and were asked to perform 10 squats. Only those who received a “Good” mark from HALE less than 3 times and whose squat posture was considered incorrect by 3 experts (each with over 5 years of experience as a trainer with a master’s degree in sports science) were allowed to participate in the study. After receiving a sufficient explanation about the experiment, the selected participants signed a consent form. The participants were divided into experimental (EXP) and control groups (CTL) using block randomization. Out of the original 34 volunteers, 29 passed the preliminary screening, and 26 passed the second screening. Thus, the study began with 13 participants in each group (experimental and control); however, 3 participants from each group dropped out in each group. Consequently, the study had a total of 20 participants (20 women), 10 participants in each group: experimental (n=10) and control (n=10). None of the participants in the data set collection were allowed to participate in this part of the study. The physical characteristics of the participants in each group are described in Table 2.

**Table 2:** Physical characteristics of the participants per group.

Characteristics	EXP^a^	CTL^b^
Age (years), mean (SD)	21.90 (2.18)	22.60 (1.95)
Height (cm), mean (SD)	163.50 (6.00)	164.70 (5.07)
Weight (kg), mean (SD)	55.32 (4.40)	50.83 (4.74)
BMI (kg/m^2^), mean (SD)	20.75 (2.11)	18.72 (1.23)
Muscle mass (kg), mean (SD)	36.87 (3.61)	35.52 (4.34)
Fat mass (kg), mean (SD)	16.03 (3.78)	12.99 (1.74)

^a^EXP: experimental group.

^b^CTL: control group.

#### Procedure

Isometric muscle function, muscle strength, and muscular endurance were measured using isokinetic equipment (Cybex 770, HUMAC NORM). Measurements were performed by measuring the extension and flexion values of the hip and knee joints. For muscle strength, angular velocity was measured at 60° per second 5 times, and muscular endurance was measured at 180° per second 15 times. The maximum torque and total workload of the knee and hip joints were also measured. All data were normalized to the participants’ body weight.

**Figure 4:**
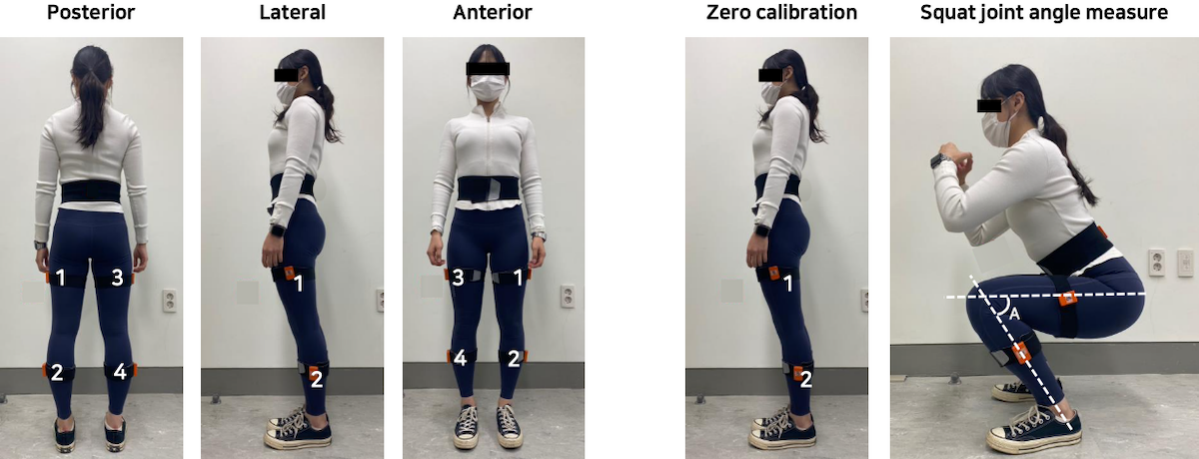
Joint angle measurement using inertial measurement unit (IMU) sensors. Angle A on the rightmost image: knee joint angle.

In addition, XSENS MTi-1 inertial measurement unit sensors (sampling at 100 Hz) were attached to identify 3D movements of the knee joints. The ROM of the left and right knee joints was calculated by combining the data from the inertial measurement unit sensors (Figure 4). Finally, participants’ body composition analysis results were recorded.

After the presession, each participant was given a smartphone with its app installed. The CTL was given a version of the app without feedback capabilities, whereas the EXP received the full version. Each group was asked to regularly practice squats using their respective apps for a predetermined amount of time, with their progress tracked through the logs left on the app server. The squat practice sessions were designed according to the American College of Sport Medicine guidelines as follows:

All participants were required to spend 30 minutes 5 days a week each session (5-minute warm-up, 20-minute main exercise, 5-minute cooldown). During the main exercise, participants performed squats for 1 minute and rested for 20-30 seconds. For the EXP, the last squat in each set was assessed as either “good” or “bad” during the resting period. The training sessions were recommended to be continuous for 30 min, but depending on the participant’s stamina and schedule, they could be split into multiple sessions; when that was the case, they were advised to have sessions no shorter than 10 min. Table 3 shows a breakdown of the training sessions.

**Table 3:** App intervention program.

Order	Type	Time	Frequency
Warm-up	Stretching (neck, shoulder, waist, legs, and ankles)	5 minutes	5 days/week
Main exercise	(60-second squat work out + ≈20-30–second break) × 15 sets	≈20-22.5 minutes	5 days/week
Cooldown	Stretching (neck, shoulder, waist, legs, and ankles)	5 minutes	5 days/week

HALE was designed to send a notification if the participant did not open the app for longer than 24 hours, with the progress of each participant checked through the logs on the server. A check-in call was given on the following day whenever a participant failed to complete the training session on any given day.

After 2 weeks of training and the final recording session, all the smartphones were collected from the participants to collect and analyze data.

### Statistical Analysis

Statistical analyses were performed on the 3 measures—the overall squat score assessed by HALE, the knee joint ROM, and the isokinetic muscle strength. Two-way mixed ANOVA [26] was used to analyze the interaction effects. Subsequently, 1-way repeated-measures ANOVA was applied for each group to analyze the effects of sessions per group. If any significant differences were detected, a post hoc comparison analysis using the least significant difference was performed.

### Ethics Approval

After receiving sufficient explanation about the first part of the experiment, participants signed a consent form approved by the Pusan University Bioethics Committee (PNU IRB/2019_38_HR), and after receiving sufficient explanation about the second part of the experiment, the selected participants signed a consent form approved by the Pusan University Bioethics Committee (PNU IRB/2020_137_HR).

## Results

As a result of Mauchly normality test [27], all items showed a normal distribution, but the CTL App test did not show normality; therefore, Greenhouse-Geisser function was used. The squat score measured by HALE was evaluated, and the results are presented in Table 4. The results of the repeated measures ANOVA for each group showed that there was a significant difference (*F*_2,18_=11.174, *P*=.001) in squat score for the EXP, while no significant effect was observed for the CTL (*F*_2,18_=2.675, *P*=.13). The post hoc analysis revealed that the participants performed significantly better during both the mid- and Post sessions than during the presession (*P*=.006 and *P*=.001, respectively). No interaction was found on the 2-way mixed ANOVA.

The results of the repeated-measures ANOVA for each group are shown in Table 5. For the EXP, there were significant differences in the left (*F*_2,18_=5.811, *P*=.02) and right (*F*_2,18_=4.736, *P*=.03) knee joint angles, while no significant effect was observed for the CTL in the left (*F*_2,18_=0.398, *P*=.68) and right (*F*_2,18_=0.517, *P*=.61) knee joint angles. Post hoc analysis revealed that the left knee joint angle in the postsession was smaller than that in the pre and midsessions (*P*=.048 and *P*=.02, respectively). In addition, the joint angle of the right knee in the postsession was smaller than that in the midsession (*P*=.02). No significant interaction was observed when 2-way mixed ANOVA was performed.

The changes in isokinetic muscle strength measured during each session were also analyzed. Regardless of the group, the participants’ muscle strength and endurance of the trunk and knees either increased significantly or showed a tendency to increase.

**Table 4:** Results of the overall squat score measured by Home Alone Exercise (HALE).

Session			Squat posture, mean (SD)		Post hoc	1–β		ES^a^			95% CI	*F*(*df*; *P* value)	
**Group EXP^b^ (N=10)**					Pre<Mid (*P*=.006);Pre<Post (*P*=.001)	.945			0.554				11.174 (2,18; .001)
	Pre	0.20 (0.42)								–0.102 to 0.502			
	Mid	4.20 (3.43)								1.750 to 6.650			
	Post	8.00 (5.20)								4.275 to 11.725			
**Group CTL^c^ (N=10)**					NS^d^	.284			0.229				2.675 (2,18; .13)
	Pre	0.70 (1.63)								–0.471 to 1.871			
	Mid	1.30 (2.50)								–0.486 to 3.086			
	Post	3.80 (5.43)								–0.086 to 7.686			

^a^ES: effect size.

^b^EXP: experimental group.

^c^CTL: control group.

^d^NS: nonsignificant; significance level at .05.

**Table 5:** Results of the joint angles.

Body		Session	Joint angle, mean (SD)	Post hoc	1–β	ES^a^	95% CI		*F* (*df*; *P* value)	
**Group EXP^b^ (N=10)**										
	**Right knee**			Mid>Post (*P*=.018)	.722	0.904			4.736 (2,18; .03)	
		Pre	71.99 (20.79)				54.612-89.383			
		Mid	76.68 (15.16)				64.011-89.364			
		Post	62.82 (14.88)				50.379-75.261			
	**Left knee**			Pre>Post (*P*=.048); Mid<Post (*P*=.02)	.822	0.454			5.811 (2,18; .02)	
		Pre	75.06 (22.16)				56.534-93.599			
		Mid	76.24 (19.37)				60.042-92.441			
		Post	63.13 (13.06)				52.213-74.064			
**Group CTL^c^ (N=10)**										
	**Right knee**			NS^d^	.061	0.054			0.517 (2,18; .61)	
		Pre	70.82 (22.71)				54.579-87.071			
		Mid	74.02 (17.55)				61.470-86.582			
		Post	70.23 (15.42)				59.200-81.274			
	**Left knee**			NS	.056	0.042				0.398 (2,18; .68)
		Pre	73.27 (23.15)				56.711-89.937			
		Mid	74.05 (17.81)				5.635-61.307			
		Post	70.70 (16.34)				5.169-59.015			

^a^ES: effect size.

^b^EXP: experimental group.

^c^CTL: control group.

^d^NS: nonsignificant; significance level at .05.

## Discussion

### Principal Results

The results of the isokinetic muscle strength test indicate that all the participants, regardless of their group, performed the squats as instructed. However, while they all gained muscle, only the participants in the EXP increased the ROM of the knee joint by 12.8% in the right leg and 15.9% in the left leg. In contrast, the ROM of the soft joint in the CTL increased by 0.9% in the right leg and by 3.6% in the left leg. The EXP showed significant improvement in the wider advanced squat technique compared to the CTL [28,29]. In addition, the results of the overall squat score and the joints’ ROM indicated that the participants who used HALE effectively improved their squat skills, whereas those who did not use HALE showed no significant improvements. The ratio of squats in the correct posture increased by 52.3% in the EXP and 21.3% in the CTL, indicating that the developed app worked properly and was effective.

Moreover, while the exercise app only provided pass-or-fail feedback along with a skeleton overlaid video playback, it effectively guided participants to learn small details about squat exercises, such as how far they had to sit down to properly perform a full squat exercise. In other words, when an automatic evaluation and self-discovery [30] system is combined with physical training programs, people can effectively develop exercise skills without direct instruction. Such findings and approaches utilizing a deep learning–based evaluation and feedback process, are expected to benefit various communities seeking to develop effective exercise programs that can be remotely taught and learned.

Furthermore, to provide advanced feedback and guidance, the size and complexity of both data sets and deep learning models must increase drastically. As the deep learning model only was needed to determine whether a trainee correctly performed an exercise, the otherwise complex problem involving 3D motion guidance became as easy as a binary classification problem. In other words, the deep learning model was able to be trained with high accuracy within a short period of time using approximately 2000 squat video data taken from the front, which simplified data labeling, processing time, and effort. In addition, the study results indicate that the participants effectively improved their squats, even though the proposed solution did not provide detailed feedback or specific guidance for improvement. Such a simplified training method possesses great potential to boost web-based or remote exercise platforms, cover various exercises, and can be expanded to remote communities.

### Limitations

Different from conventional training systems, HALE does not provide direct instructions. However, while such an approach may initially be mistaken as more difficult to learn, the study results indicate that the users experienced more effective workout sessions using the app even without direct instructions. In fact, the indirect feedback was able to help the users to engage and learn by themselves about the correct postures as they tried to improve their scores, suggesting that machine learning models can be trained effectively as training tools, even with minimal data and simple architectures. Choosing more complex model architectures, collecting more data to improve the model, or providing more detailed instructions to users might be viable options, but those approaches could be too costly compared to the benefits and do not even guarantee better teaching effects.

In addition, HALE might lack robustness; the user must place the smartphone at a predetermined distance, height, and angle, or the app cannot correctly assess their squat. However, this issue can be resolved by using the recently developed 3D pose estimation algorithms [31]. The current version of HALE only supports squats; however, it can be extended to other exercises by collecting new data sets and training new models.

Unfortunately, it was difficult to find more appropriate participants who had little or no experience with squats because most men had some exercise experience. However, while further investigation on additional men might be beneficial, the difference is likely to be marginal, as evidenced by prior work, which indicated no significant difference between men’s and women’s exercise efficacy [32,33].

### Conclusions

This study demonstrated the effectiveness of a deep learning-based personal workout assistant that can provide feedback on squat postures using only mobile devices. In the first part of the study, the squat video data set was created and the deep learning model, which showed a test accuracy of 85%, was trained. In the second part, the mobile workout assistant app, HALE, was developed and the interventional study showed how it helped people improve squat exercise. As demonstrated by the improvements in the squat posture and joint ROM, the EXP trained with HALE experienced faster improvement and learned more nuanced details of the squat exercise. The proposed mobile app that is low cost and provides self-discovery feedback effectively taught users about squat exercises without expensive in-person sessions with a trainer.

## Appendix

Multimedia Appendix 1 CONSORT-eHEALTH checklist (V 1.6.1).

## Abbreviations

CTL: control group
EXP: experimental group
HALE: home alone exercise
LSTM: long short-term memory
RGB: red green blue
ROM: range of motion
